# Melt-Crystallizations of α and γ Forms of Isotactic Polypropylene in Propene-Butene Copolymers

**DOI:** 10.3390/polym14183873

**Published:** 2022-09-16

**Authors:** Miriam Scoti, Fabio De Stefano, Filomena Piscitelli, Giovanni Talarico, Angelo Giordano, Claudio De Rosa

**Affiliations:** Dipartimento di Scienze Chimiche, Università di Napoli Federico II, Complesso Monte S. Angelo, Via Cintia, 80126 Napoli, Italy

**Keywords:** propylene-butene copolymers, α and γ forms, metallocene catalysts, melt-crystallization

## Abstract

Random isotactic propene-butene copolymers (iPPC4) of different stereoregularity have been synthesized with three different homogeneous single center metallocene catalysts having different stereoselectivity. All samples crystallize from the polymerization solution in mixtures of α and γ forms, and the relative amount of γ form increases with increasing concentrations of butene and of *rr* stereodefects. All samples crystallize from the melt in mixtures of α and γ forms and the fraction of γ form increases with decreasing cooling rate. At high cooling rates, the crystallization of the α form is always favored, even for samples that contain high total concentration of defects that should crystallize in the γ form. The results demonstrate that in iPPs containing significant concentrations of defects, such as stereodefects and comonomeric units, the γ form is the thermodynamically stable form of iPP and crystallizes in selective conditions of very slow crystallization, whereas the α form is the kinetically favored form and crystallizes in conditions of fast crystallization.

## 1. Introduction

The crystallization of α and γ forms of isotactic polypropylene (iPP) depends on the conditions of crystallization and on the molecular structure of polypropylene; the latter depends on the polymerization conditions and catalysis [1]. Different conditions of polymerization and used catalysts produce iPP macromolecules characterized by different molecular structures, because different catalysts may introduce different types and amounts of microstructural defects, such as defects of stereoregularity and regioregularity, constitutional defects and different distribution of defects along the chains [1,2,3,4,5].

The α form is considered the most stable form of iPP and crystallizes usually in the iPP homopoymer prepared with heterogeneous Ziegler-Natta catalysts in common crystallization conditions from the melt or from solution, and in stretched fibers [6,7,8]. In the same commercial iPP samples the γ form crystallizes only in special conditions, as in samples of low molecular mass [9,10,11,12,13] and by crystallization at high pressures [14,15,16,17,18]. A low amount of γ form has also been obtained in some copolymers of propylene with different comonomers synthesized with the same heterogeneous catalysts [19,20,21,22,23,24,25,26,27,28,29,30,31,32,33,34,35,36,37,38,39,40,41,42,43,44,45,46,47]. The γ form crystallizes, instead, easily in iPP samples [48,49,50,51,52,53,54] and its copolymers [55,56,57,58,59,60,61,62,63,64,65,66,67,68,69,70,71,72,73,74,75,76,77,78,79,80,81,82,83,84,85,86,87,88,89,90,91,92] synthesized with homogeneous single site metallocene catalysts [2,3,4,5], which introduce different kinds of defects depending on the catalyst structure. These defects, as stereo-defects, regio-defects and also comonomers, indeed, favors crystallization of the γ form [48,49,50,51,52,53,54,55,56,57,58,59,60,61,62,63,64,65,66,67,68,69,70,71,72,73,74,75,76,77,78,79,80,81,82,83,84,85,86,87,88,89,90,91,92]. 

The crystallization of the γ form in chains containing defects is due to the fact that the γ form crystallizes when iPP chains are characterized by short regular propene sequences, therefore, it occurs when iPP chains contain any type of defect that interrupts and shortens the regular propene sequences [51,54,62,79]. In particular, iPPs and copolymers produced with homogeneous single site metallocene catalysts are characterized by a perfectly random distribution of defects along the macromolecules, which, therefore, shortens the length of the regular propene sequences even for low concentrations of defects [51,54,62,79].

The crystallization of α form is, instead, favored when the regular propene sequences are very long, which are generated when the concentration of defects is low or when defects are segregated in blocks of the macromolecules [51,54,62,79], as, for instance, in samples synthesized with Ziegler-Natta catalysts, where the non-random distribution of defects and their segregation in blocks make the regular propene sequences always long even in the case of a high concentration of defects and in copolymers with relatively high comonomer concentration [93,94,95,96]. 

Understanding the conditions of crystallization of α and γ forms of iPP is of particular relevance since the two different polymorphs exhibit significant differences in mechanical behavior [7,46,47,54,76,77,79,87,88,89,90,91,97,98,99,100,101]. In general, the molecular architecture and topology of copolymers, from standard random to block or multiblock copolymers, greatly affects the crystallization behavior and properties of polymers because of the different length of crystallizable sequences [93,94,95,96,102,103]. While the effect of the molecular structure and architecture on the crystallization behavior of iPP has been extensively investigated and the effect of different kinds of defects on the crystallization of α and γ forms has been clarified, the effect of the conditions of crystallization is still unclear. 

In this paper we report a study of the crystallization of α and γ forms of iPP in propene-butene copolymers in different crystallization conditions to analyze the thermodynamic and kinetics effects on the crystallization of the two polymorphic forms. Propene-butene copolymers of different stereoregularity synthesized with different metallocene catalysts have been chosen for this study because they crystallize easily in the γ form, thanks to the incorporation in the iPP chains of butene comonomeric units that shorten the length of the regular propene sequences [79]. Hence, in this system, the γ form is the thermodynamically stable form of iPP and, therefore, these copolymers represent the ideal system to study the crystallization conditions that may favor crystallization of the α form.

## 2. Materials and Methods

**Scheme 1:**
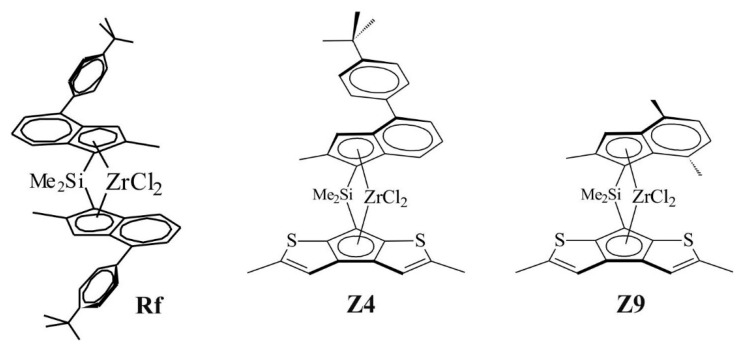
Structures of the isoselective C2-symmetric (Rf) and less isoselective C1-symmetric (Z4 and Z9) metallocene catalysts used for the synthesis of iPPC4 copolymers.

Propylene-butene isotactic copolymers (iPPC4) were synthesized with the metallocene catalysts of Scheme 1 having different stereoselectivity activated with methylalumoxane (MAO) (from Lanxess, Cologne, Germany) [79]. All operations were performed under nitrogen by using conventional Schlenk-line techniques. Toluene solvent was purified by degassing with N_2_ and passing over activated Al_2_O_3_ (8 h, N_2_ purge, 300 °C), and stored under nitrogen. The MAO cocatalyst was used as received (10 wt.%/vol. toluene solution, 1.7 M in Al). The catalyst mixture was prepared by dissolving the desired amount of the metallocene with the proper amount of the MAO solution, obtaining a solution which was stirred for 10 min at 25 °C before being injected into the reactor. All copolymerizations were run at 25 °C in a 250 mL Pyrex reactor, agitated with magnetic stirrer, containing toluene (100 mL) and MAO (2.0 mL). Gas mixtures of propene and 1-butene at the appropriate composition, prepared with vacuum line techniques in a gas cylinder at pressure of 4–5 bar and standardized by gas chromatography, were bubbled through the liquid phase at atmospheric pressure and a flow rate of 0.3 L/min. The polymerizations started by syringing in the toluene solution of the catalyst (2–3 mg) and proceeded under a constant flow of the gas mixture. Under such conditions, total monomer conversions were lower than 15%, this ensuring a nearly constant feeding ratio. The copolymers were coagulated with excess methanol acidified with enough HCl (aqueous, concentrated) to prevent the precipitation of alumina from MAO hydrolysis, filtered, washed with further methanol, and vacuum-dried. Typical yields were 2–5 g with a 120 min reaction time. 

The C_2_-symmetric metallocene Rf is highly isospecific [104,105] and produces highly isotactic iPPC4 copolymers (samples iPPC4Rf-x, where x is the butene concentration) containing negligible amounts of stereodefects (lower than 0.1 mol% of *rr* triads) and small amount of regiodefects around 0.1–0.5 mol%, represented by secondary 2,1-erythro units (2,1e). The two C_1_-symmetric metallocenes Z4 and Z9 are instead fully regioselective but introduce significant amounts of *rr* stereodefects [106,107,108,109]. Samples of iPPC4 copolymers synthesized with the catalyst Z4 (sample iPPC4Z4-x) are fully regioregular and contain 2.0–2.4 mol% of *rr* stereodefects, whereas samples synthesized with the catalyst Z9 (samples iPPC4Z9-x) contain 2.5–3.4 mol% of *rr* defects [107,108]. Consequently, the less isotactic samples synthesized with catalysts Z4 and Z9 show melting temperatures lower than those of the samples synthesized with the catalyst Rf [79]. The composition, the melting temperatures and the molecular mass of all samples are shown in Table 1. 

The composition and comonomer distribution were determined by ^13^C NMR analysis (Table 1). All spectra were obtained using a Bruker DPX-400 spectrometer operating in the Fourier transform mode at 120 °C at 100.61 MHz (Bruker Company, Billerica, MA, USA). The samples were dissolved with 8% wt/v concentration in 1,1,2,2-tetrachloroethane-d_2_ at 120 °C. The carbon spectra were acquired with a 90° pulse and 15 s of delay between pulses and CPD (WALTZ 16) to remove ^1^H-^13^C coupling. About 1500–3000 transients were stored in 32K data points using a spectral window of 6000 Hz. For all copolymer samples, the peak of the propylene methine carbon atoms was used as internal reference at 28.83 ppm. The ^13^C NMR spectra of two samples of iPPC4 copolymers are reported in Figure S1 of the Supplementary Material. The resonances were assigned according to ref. [110] and the butene concentrations in the copolymers were evaluated from the concentrations of the constitutional diads PP, PB, BB (P = propene, B = butene), using the Equations S1–S5 of the Supplementary Material. The NMR analysis showed that all the copolymers present a random distribution of comonomers and homogeneous intermolecular composition with *r*_P_ × *r*_B_ ≈ 1, calculated using the Equation S6 of the Supplementary Material, according to ref. [111]. 

The molecular masses and the dispersity were determined by gel permeation chromatography (GPC), using a Polymer Laboratories GPC220 apparatus equipped with a differential refractive index (RI) detector and a Viscotek 220R viscometer (Agilent Company, Santa Clara, CA, USA), on polymer solutions in 1,2,4-trichlorobenzene at 135 °C of 2 mg/mL concentration. The injection volume was 300 μL with a flow rate of 1.0 mL/min. The GPC apparatus was calibrated with 12 standard samples of polystyrene having narrow dispersity and molecular masses in the range 580 and 13.2 × 10^6^. 

The calorimetry measurements were carried out with differential scanning calorimeter (DSC) Mettler Toledo DSC-822 (Columbus, OH, USA) performing scans in a flowing N_2_ atmosphere and scanning rate of 10 °C/min.

X-ray powder diffraction profiles were recorded with Ni filtered Cu Kα radiation by using an Empyrean diffractometer (Malvern Panalytical, Worcestershire, UK).

All samples of iPPC4 copolymers were crystallized in DSC by cooling the melt at 180 °C down to 25 °C at different cooling rates from 1 to 40 °C/min. After the crystallization in DSC, the samples were analyzed by X-ray diffraction.

In samples that crystallized in mixtures of α and γ forms, the relative fraction of the γ form *f*_γ_, with respect to the α form, was calculated from the intensities of the (117)_γ_ and (130)_α_ reflections at 2θ = 20.1° of the γ form and at 2θ = 18.6° of the α form, respectively, as the ratio: $f_{\gamma }=I{\left(117\right)}_{\gamma }/[I{\left(117\right)}_{\gamma }+I\left(130{)}_{\alpha }\right]$.

## 3. Results and Discussion

**Figure 1:**
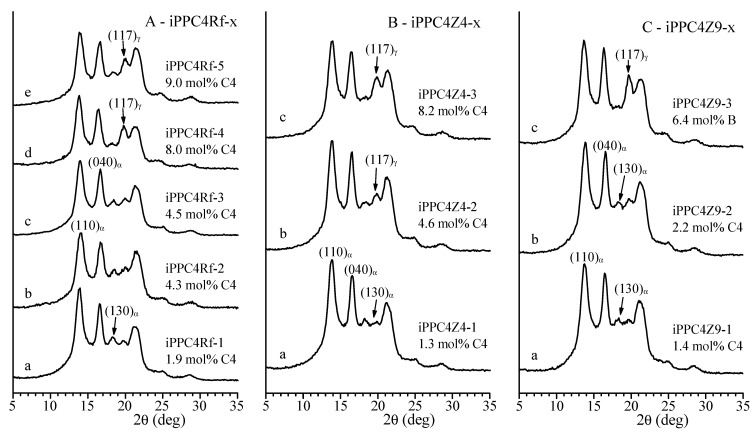
X-ray powder diffraction profiles of as-prepared samples of iPPC4 copolymers of the indicated butene concentration. Isotactic samples iPPC4Rf-x with [*rr*] < 0.1 mol% (**A**) and less isotactic samples iPPC4Z4-x with [*rr*] = 2.0–2.4 mol% (**B**) and iPPC4Z9-x with [*rr*] = 2.5–3.4 mol% (**C**).

**Figure 2:**
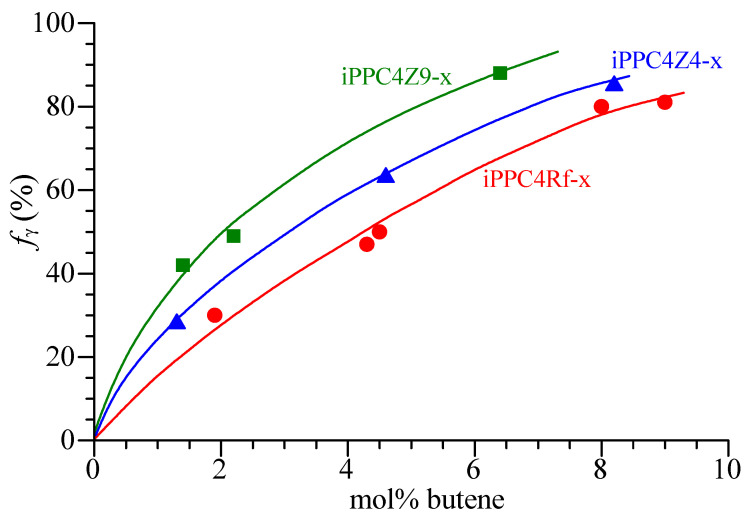
Values of the fraction of γ form that crystallizes in the as-prepared samples of iPPC4 copolymers as a function of butene concentration evaluated from the diffraction profiles of Figure 1. Isotactic samples iPPC4Rf-x with [*rr*] < 0.1 mol% (●) and less isotactic samples iPPC4Z4-x with [*rr*] = 2.0–2.4 mol% (▲) and iPPC4Z9-x with [*rr*] = 2.5–3.4 mol% (■).

The X-ray diffraction profiles of as-prepared (precipitated from the polymerization solution) samples of iPPC4 copolymers of Table 1 are reported in Figure 1. The diffraction profiles present the (130)_α_ and (117)_γ_ reflections at 2θ = 18.6 and 20.1° of the α and γ forms, respectively, indicating that all as-prepared samples of iPPC4 copolymers are crystallized in mixtures of α and γ forms. The intensity of the (117)_γ_ reflection increases with increasing butene concentration and, at the same butene content, is higher in the low stereoregular samples synthesized with the catalysts Z4 and Z9 (samples iPPC4Z4-x and iPPC4Z9-x, Figure 1B,C). The values of the relative amount of γ form (*f*_γ_) calculated from the intensities of the (117)_γ_ and (130)_α_ reflections in the diffraction profiles of Figure 1 are reported in Figure 2 as a function of butene concentration. Since the highly stereoregular iPP homopolymer generally crystallizes in the α form, it has been assumed *f*_γ_ = 0 for butene concentration equal to zero. It is apparent that the amount of γ form increases with increasing concentration of butene and of *rr* stereodefects [79] (Figure 2). The more isotactic samples iPPC4Rf-x crystallize almost in the pure γ form (*f*_γ_ ≈ 80%) for butene concentration of nearly 9 mol% (sample iPPC4Rf-5, profile e in Figure 1A and Figure 2), whereas for the less isotactic samples iPPC4Z9-x, the highest amount of γ form (*f*_γ_ ≈ 88%) crystallizes at about 6 mol% of butene (sample iPPC4Z9-3, profile c in Figure 1C and Figure 2). 

It is worth reminding that in iPPC4 copolymers the further increase of butene concentration induces decrease of the amount of γ form and crystallization of the α form for concentrations higher than 15–20 mol% because butene units are included easily in the crystals of α form producing stabilization of the α form compared to the γ form [79].

**Figure 3:**
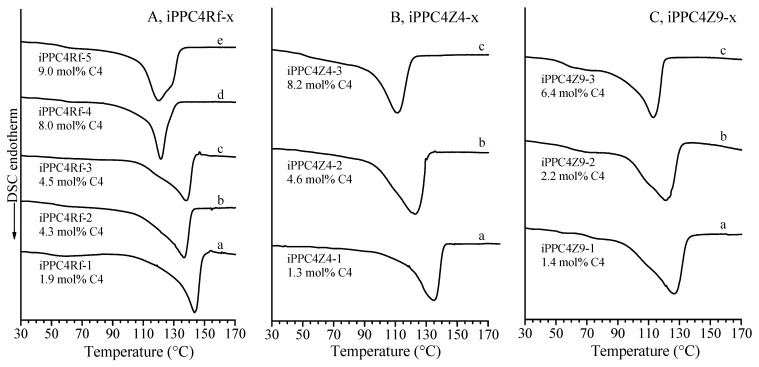
DSC heating curves recorded at 10 °C/min of as-prepared samples of iPPC4 copolymers of the indicated butene concentration. Isotactic samples iPPC4Rf-x with [*rr*] < 0.1 mol% (**A**) and less isotactic samples iPPC4Z4-x with [*rr*] = 2.0–2.4 mol% (**B**) and iPPC4Z9-x with [*rr*] = 2.5–3.4 mol% (**C**).

**Figure 4:**
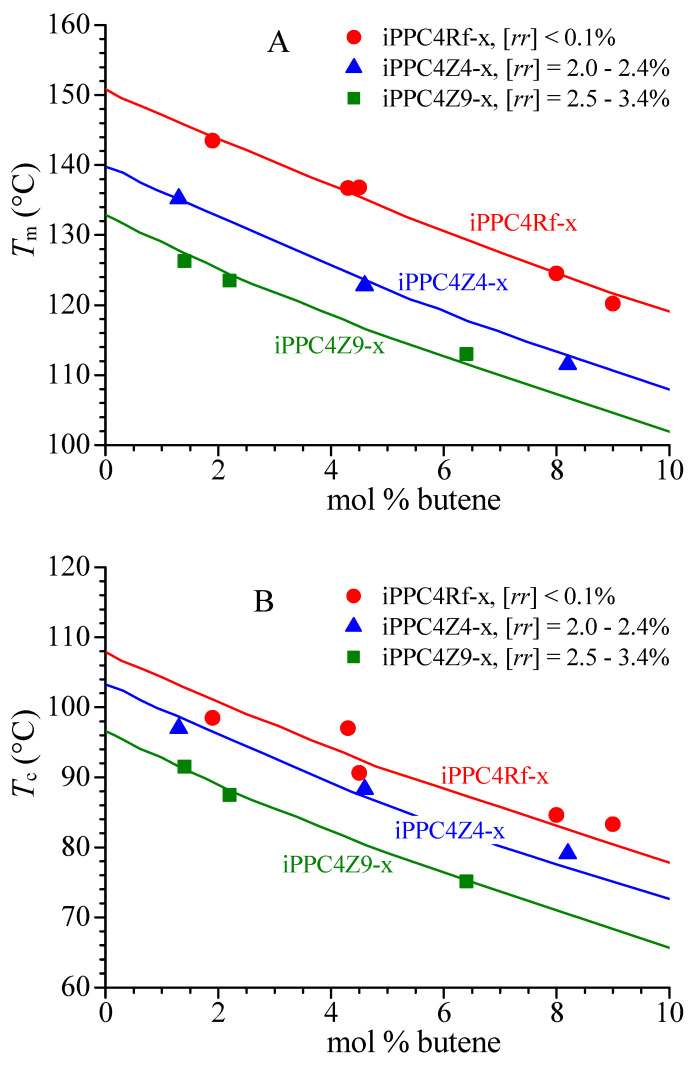
Melting temperature of the as-prepared samples (**A**) and crystallization temperature from the melt (**B**) of iPPC4 copolymers as a function of butene concentration evaluated from DSC thermograms recorded at scanning rates of 10 °C/min. Isotactic samples iPPC4Rf-x with [*rr*] < 0.1 mol% (●) and less isotactic samples iPPC4Z4-x with [*rr*] = 2.0–2.4 mol% (▲) and iPPC4Z9-x with [*rr*] = 2.5–3.4 mol% (■).

The DSC heating curves of the as-polymerized samples of iPPC4 copolymers are reported in Figure 3. For the three sets of samples the melting temperature decreases with increasing butene concentration. The values of the melting temperature are reported in Table 1 and in Figure 4A as a function of butene concentration.

It is apparent from Figure 4A that the melting temperature also depends on the stereoregularity of the samples and on the concentration of *rr* defects. In fact, at the same butene concentration the more isotactic samples iPPC4Rf-x show melting temperatures higher than those of the less isotactic samples iPPC4Z4-x and iPPC4Z9-x, and the samples iPPC4Z9-x with the highest concentration of *rr* stereodefects show the lowest melting temperatures.

**Figure 5:**
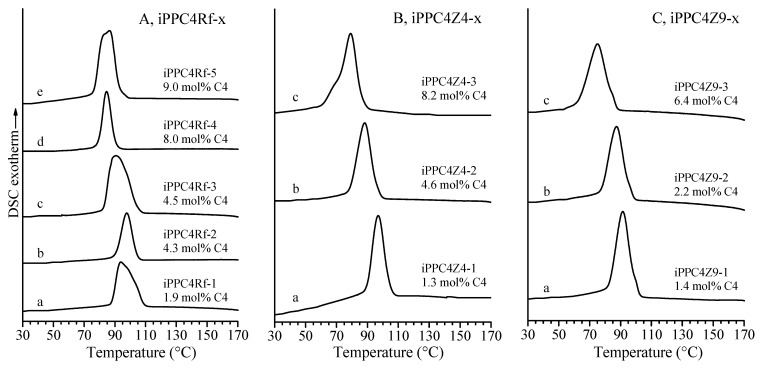
DSC cooling curves recorded at 10 °C/min of samples of iPPC4 copolymers of the indicated butene concentration after melting at 170 °C (Figure 3). Isotactic samples iPPC4Rf-x with [*rr*] < 0.1 mol% (**A**) and less isotactic samples iPPC4Z4-x with [*rr*] = 2.0–2.4 mol% (**B**) and iPPC4Z9-x with [*rr*] = 2.5–3.4 mol% (**C**).

All samples have been crystallized in DSC from the melt by cooling at different cooling rates. The samples have been melted by heating at 10 °C/min up to 170 °C, as in Figure 3, and then cooled from 170 °C down to 25 °C at different cooling rates, from 1 °C/min to 40 °C/min. As an example, the DSC cooling curves of all samples recorded at cooling rate of 10 °C/min are reported in Figure 5. All samples crystallize during cooling and the DSC curves of Figure 5 show well-defined exothermic peaks. The values of the crystallization temperature evaluated from the DSC cooling curves of Figure 5 at cooling rate of 10 °C/min are plotted in Figure 4B as a function of butene concentration. For the three sets of samples, the crystallization temperature decreases with increasing butene concentration and, at the same butene concentration, decreasing the stereoregularity (Figure 4B). Therefore, introduction of both butene co-units and *rr* stereodefects produces a decrease of melting and crystallization temperatures (Figure 4) and an increase of the fraction of γ form (Figure 2).

**Figure 6:**
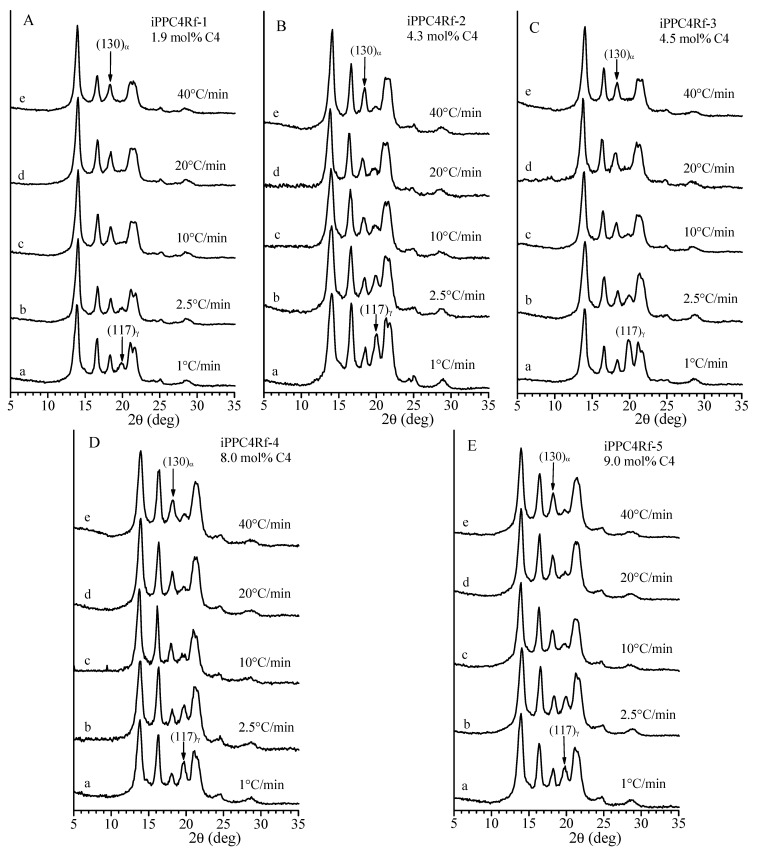
X-ray diffraction profiles of samples of copolymers iPPC4Rf-x crystallized from the melt by cooling the melt from 170 °C down to 25 °C at the indicated different cooling rates.

**Figure 7:**
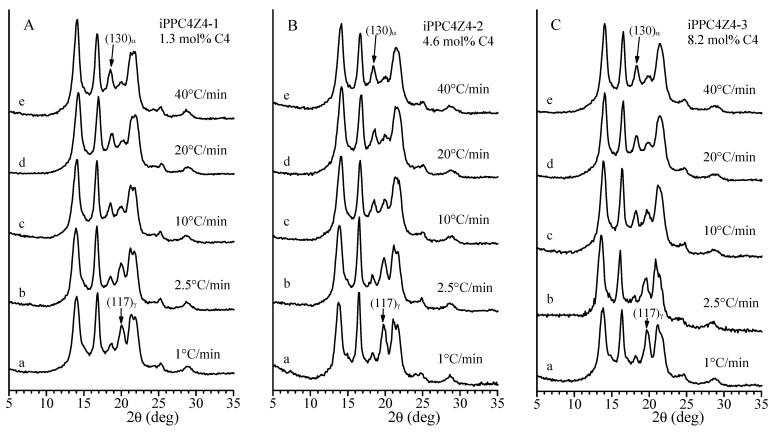
X-ray diffraction profiles of samples of copolymers iPPC4Z4-x crystallized from the melt by cooling the melt from 170 °C down to 25 °C at the indicated different cooling rates.

**Figure 8:**
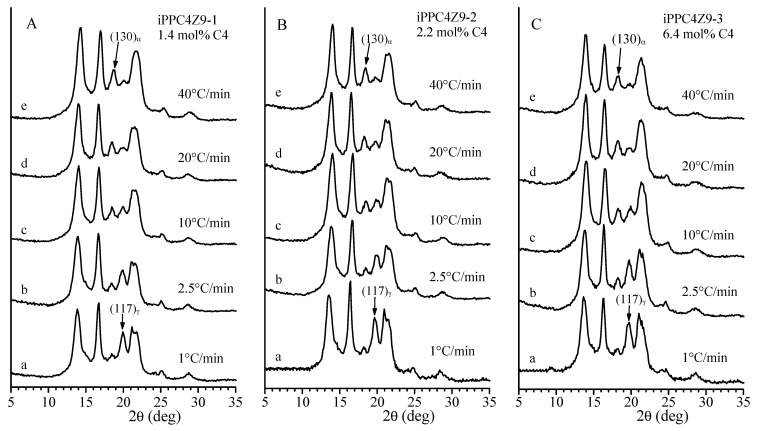
X-ray diffraction profiles of samples of copolymers iPPC4Z9-x crystallized from the melt by cooling the melt from 170 °C down to 25 °C at the indicated different cooling rates.

After the crystallization in DSC as in Figure 5, the samples were analyzed by X-ray diffraction at 25 °C. The diffraction profiles of the samples iPPC4Rf-x, iPPC4Z4-x and iPPC4Z9-x crystallized from the melt at different cooling rates are reported in Figure 6, Figure 7 and Figure 8, respectively. All samples crystallize into mixtures of α and γ forms and the amounts of the two forms strongly depend on the cooling rate.

**Figure 9:**
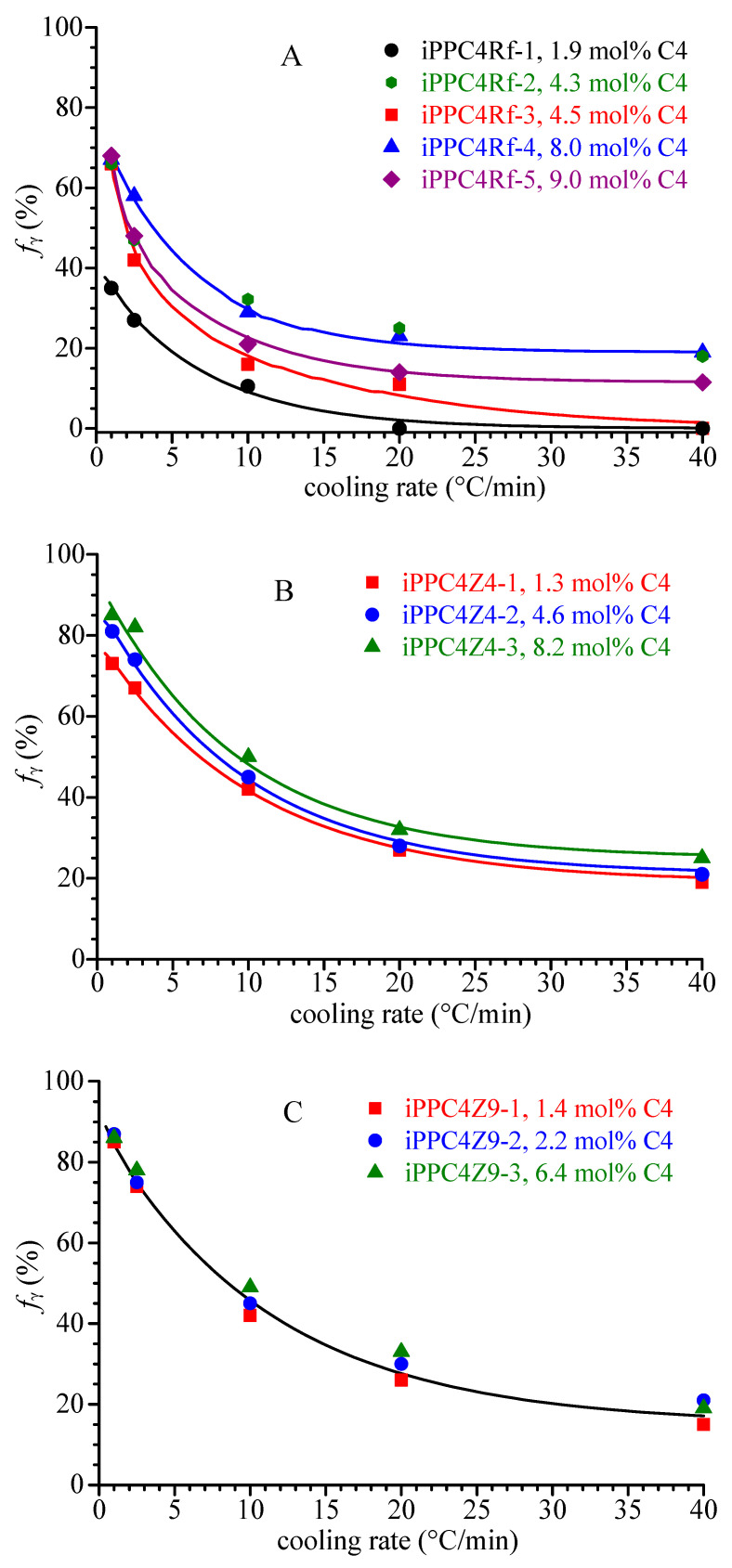
Values of the fraction of γ form that crystallizes in samples of iPPC4 copolymers cooled from the melt at different cooling rates as a function of the cooling rate, evaluated from the diffraction profiles of Figure 6, Figure 7 and Figure 8. (**A**) Isotactic samples iPPC4Rf-x with [*rr*] < 0.1 mol%, (**B**) samples iPPC4Z4-x with [*rr*] = 2.0–2.4 mol%, and (**C**) samples iPPC4Z9-x with [*rr*] = 2.5–3.4 mol%.

The samples displayed the same behavior regardless of the butene concentration and stereoregularity, that is, the intensity of the (130)_α_ reflection of the α form at 2θ = 18.6° decreases and the intensity of the (117)_γ_ reflection at 2θ = 20.1° of the γ form increases with decreasing cooling rate. The amount of γ form is reported in Figure 9 as a function of the cooling rate for the three sets of samples. These data indicate that the amount of γ form increases with decreasing cooling rate. Correspondingly, the γ form almost disappears and the almost pure α form crystallizes in all samples at the highest cooling rate of 40 °C/min (profiles e of Figure 6, Figure 7 and Figure 8). The highest concentration of γ form is always obtained at low cooling rates, whereas at high cooling rates the crystallization of the α form is always favored, even for samples that contain high total concentration of defects (high butene and high *rr* defects concentrations) that tend to crystallize normally in the γ form. In fact, even the less isotactic samples iPPC4Z4-3 and iPPC4Z9-3 with the highest butene concentrations of 8.2 and 6.4 mol%, respectively, that crystallize in the as-prepared samples in the almost pure γ form (*f*_γ_ = 85 and 88%, respectively) (profiles c of Figure 1B,C and Figure 2), crystallize in the α form in the fast crystallization from the melt at high cooling rate (profiles e of Figure 7 and Figure 8).

These results demonstrate that in iPP samples containing significant concentrations of defects, such as stereodefects and butene comonomeric units, the γ form is the thermodynamically stable form of iPP and crystallizes normally from polymer solution or in selective conditions of very slow crystallization, as isothermal crystallizations from the melt at high crystallization temperatures [79] or by cooling from the melt at very low cooling rates, whereas the α form is the kinetically favored form and crystallizes in conditions of fast crystallization, such as fast cooling from the melt.

## 4. Conclusions

Random isotactic propene-butene copolymers of different stereoregularity have been prepared with three different metallocene catalysts having different stereoselectivity. The C_2_-symmetric metallocene produces highly isotactic iPPC4 copolymers containing negligible amounts of stereodefects, whereas the two C_1_-symmetric metallocenes produce less isotactic copolymers containing on average 2.0–2.4 mol% and 2.5–3.4 mol% of *rr* stereodefects, respectively.

All as-prepared samples crystallize in mixtures of α and γ forms from the polymerization solution and the relative amount of γ form increases with increasing concentrations of butene and of *rr* stereodefects.

The samples have been crystallized from the melt by cooling the melt from 170 °C down to 25 °C at different cooling rates. All samples crystallize from the melt in mixtures of α and γ forms and the amount of the two forms strongly depends on the cooling rate. The amount of γ form increases with decreasing cooling rate. Correspondingly, the γ form almost disappears and the almost pure α form crystallizes in all samples at the highest cooling rate of 40 °C/min. The highest concentration of γ form is always obtained at low cooling rates, whereas at high cooling rates the crystallization of the α form is always favored, even for samples that contain high total concentration of defects that should crystallize in the γ form.

These results demonstrate that in iPP samples containing significant concentrations of defects, such as stereodefects and butene comonomeric units, the γ form is the thermodynamically stable form of iPP and crystallizes normally from polymer solution or in selective conditions of very slow crystallization, such as by cooling from the melt at very low cooling rates, whereas the α form is the kinetically favored form and crystallizes in conditions of fast crystallization, such as fast cooling from the melt.

## Figures and Tables

**Table 1:** Catalyst, composition (mol% butene), melting temperature of as-prepared samples (*T*_m_), molecular mass (*M*_w_) and dispersity (*M*_w_/*M*_n_) of the iPPC4 copolymers [79].

Sample	Catalyst	mol% Butene	*T*_m_ (°C) ^a^	*M*_w_^b^	*M*_w_/*M*_n_^b^
iPPC4Rf-1	Rf	1.9	143	316,500	2.2
iPPC4Rf-2	Rf	4.3	137	228,700	2.1
iPPC4Rf-3	Rf	4.5	137	207,000	2.0
iPPC4Rf-4	Rf	8.0	125	178,500	2.0
iPPC4Rf-5	Rf	9.0	120	200,000	2.1
iPPC4Z4-1	Z4	1.3	135	172,900	2.1
iPPC4Z4-2	Z4	4.6	123	175,700	2.0
iPPC4Z4-3	Z4	8.2	112	176,700	2.0
iPPC4Z9-1	Z9	1.4	126	214,000	2.1
iPPC4Z9-2	Z9	2.2	124	214,500	2.0
iPPC4Z9-3	Z9	6.4	113	214,400	2.0

^a^ Determined from DSC heating curves recorded at 10 °C/min. ^b^ Determined by GPC.

## Data Availability

The data in this study are available on reasonable request from the corresponding author.

## Supplementary Materials

The following supporting information can be downloaded at: https://www.mdpi.com/article/10.3390/polym14183873/s1, Figure S1: ^13^C NMR spectra of the iPPC4 copolymer samples iPPC4Rf-1 with 1.9 mol% of butene and iPPC4Rf-5 with 9.0 mol% of butene; Equations (S1)–(S5) used for the calculation of the concentrations of the constitutional diads PP, PP and BB (P = propene, B = butene) and the concentration of the comonomeric units from the ^13^CNMR data; Equation S6 used for the calculation of the product of reactivity ratios *r*_P_ × *r*_B_. References [110,111] are cited in the supplementary materials.
