# Supplementary material for: Melt-Crystallizations of α and γ Forms of Isotactic Polypropylene in Propene-Butene Copolymers
Source: Polymers (Basel). 2022 Sep 16;14(18):3873. doi: 10.3390/polym14183873 (PMC9500696; doi:10.3390/polym14183873)
Supplement: Supplementary file 1 [file polymers-14-03873-s001.zip › polymers-1911817-supplementary.pdf]

Supplementary Material for the paper:

Melt-Crystallizations of α and γ Forms of Isotactic Polypropylene in Propene-Butene Copolymers

Miriam Scoti,* Fabio De Stefano, Filomena Piscitelli,† Giovanni Talarico, Angelo Giordano, Claudio De Rosa*

Dipartimento di Scienze Chimiche, Università di Napoli Federico II, Complesso Monte S. Angelo, Via Cintia, 80126 Napoli, Italy.

†Present Address: Department of Materials and Structures, CIRA–Italian Aerospace Research Centre, Via Maiorise, 81043 Capua, Italy

1. ^{13}C NMR spectra

The microstructures of all samples of propene-butene copolymers (iPPC4) have been studied with ^{13}C NMR spectroscopy. All spectra were obtained using a Bruker DPX-400 spectrometer operating in the Fourier transform mode at 120 °C at 100.61 MHz (Bruker Company, Billerica, Massachusetts, USA). The samples were dissolved with 8% wt/v concentration in 1,1,2,2-tetrachloroethane-d_2 at 120 °C. The carbon spectra were acquired with a 90° pulse and 15 seconds of delay between pulses and CPD (WALTZ 16) to remove ^{1}H-^{13}C coupling. About 1500-3000 transients were stored in 32K data points using a spectral window of 6000 Hz. For all copolymer samples, the peak of the propylene methine carbon atoms was used as internal reference at 28.83 ppm. The resonances were assigned according to ref. [110] of the main text references and the butene concentrations in the copolymers were evaluated from the concentrations of the constitutional diads PP, PB, BB (P = propene, B = butene). The concentrations of diads PP, PB and BB and the composition were obtained from the $S_{\alpha\alpha}$ peaks using the following equations:

$$BB = 100S_{\alpha\alpha}(BB)/\Sigma S_{\alpha\alpha} \quad (S1)$$
$$PB = 100S_{\alpha\alpha}(PB)/\Sigma S_{\alpha\alpha} \quad (S2)$$
$$PP = 100S_{\alpha\alpha}(PP)/\Sigma S_{\alpha\alpha} \quad (S3)$$
$$[P] = PP+0.5PB \quad (S4)$$
$$[B] = BB+0.5PB \quad (S5)$$

The product of reactivity ratios $r_P{\times}r_B$ was determined from the diads according to ref. [111] of the main text references as:

$$r_P{\times}r_B = 4[PP]\times[BB]/[PB]^2 \quad (S6)$$

The NMR analysis showed that all the copolymers present a statistical distribution of comonomers and homogeneous intermolecular composition with $r_P \times r_B \approx 1$.
The ^{13}C NMR spectra of some samples of iPPC4 copolymers showing the resonances assigned to the butene (B) are reported in Figure S1.

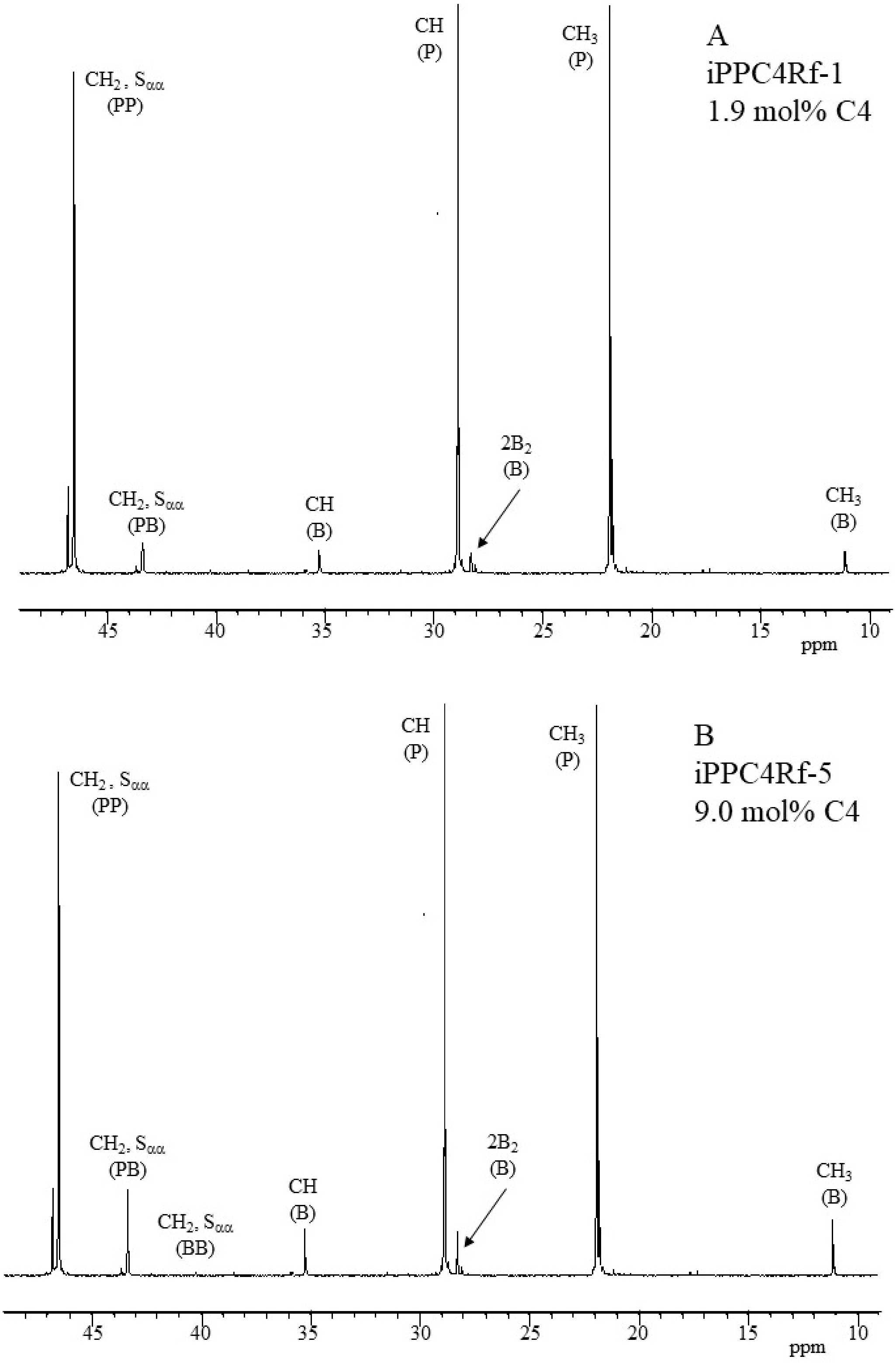

Figure S1. ^{13}C NMR spectra of the iPPC4 copolymer samples iPPC4Rf-1 with 1.9 mol% of butene (A) and iPPC4Rf-5 with 9.0 mol% of butene (B). Scale is in ppm downfield of TMS.

References

110. Randall J. C. A ^{13}C NMR Determination of the Comonomer Sequence Distributions in Propylene-Butene-1 Copolymers *Macromolecules* **1978**, *11*, 592.
111. Kakugo, M.; Naito, Y.; Mizunuma, K.; Miyatake, T. Carbon-13 NMR determination of monomer sequence distribution in ethylene-propylene copolymers prepared with δ-$TiCl_3$-$Al(C_2H_5)_2Cl$. *Macromolecules* **1982**, *15*, 1150.
